# Anatomical Variations of Lumbar Arteries and Their Clinical Implications: A Cadaveric Study

**DOI:** 10.5402/2013/154625

**Published:** 2013-09-12

**Authors:** Aranjan Lionel Karunanayake, Arunasalam Pathmeswaran

**Affiliations:** ^1^Department of Anatomy, Faculty of Medicine, University of Kelaniya, 0094 11 Ragama, Sri Lanka; ^2^Department of Public Health, Faculty of Medicine, University of Kelaniya, 0094 11 Ragama, Sri Lanka

## Abstract

Lumbar arteries arise from the abdominal aorta. Some abdominal and spinal surgeries can damage these arteries, and that can lead to serious consequences. This study aimed at studying the types and frequencies of variations of lumbar vasculature. We dissected both sides of 109 adult human cadavers and studied the variations of lumbar vasculature. Age range was 43–90 years. Fifty-seven percent were males and 43% were females. The number of lumbar arteries arising from either side of the abdominal aorta varied between 3 and 5 pairs. The lumbar arteries arose from a common single stem in 12% of the cadavers. The third and fourth pairs of lumbar arteries arose from a common single stem in 3% and 11% of cadavers, respectively, and the first and second pairs of lumbar arteries arose from a common single stem in 1% and 2% of cadavers, respectively. The first and second lumbar arteries on the right side traveled anterior to the right crus of the diaphragm in 7% and 8% of cadavers, respectively. There were several variations with regard to the number, origin from the abdominal aorta, and pathway of lumbar arteries from what is described in the literature.

## 1. Introduction 

There are four lumbar arteries on the right and left sides of the body, which arise from the posterolateral aspect of the abdominal aorta opposite the four lumbar vertebrae. A fifth smaller pair may occasionally arise from the median sacral artery, but lumbar branches of iliolumbar arteries usually take their place [1]. The median sacral artery arises from the posterior aspect of the abdominal aorta just above the aortic bifurcation, and the iliolumbar artery arises from the common iliac artery [1]. According to Glodny et al., iliolumbar artery can arise from the abdominal aorta [2]. The lumbar arteries run posterolaterally on the upper four lumbar vertebral bodies, pass posterior to the sympathetic trunks into the gaps between the lumbar transverse processes, and continue into the muscles of the abdominal wall [1]. The arteries of the right side pass posterior to the inferior vena cava (IVC). The first two lumbar arteries on the right side and the first lumbar artery on the left side run posterior to the right and left crura of the diaphragm, respectively [1]. These arteries anastomose with each other and also with lower posterior intercostals, subcostal, iliolumbar, deep circumflex iliac, and inferior epigastric arteries. Along their path from the origin to the final destination, the said arteries supply the muscles, fasciae, ligaments, intervertebral discs, vertebrae, and joints [1]. Knowing the anatomy of lumbar arteries is important to understand many clinical problems related to the lumbar region and to use them in many surgical procedures done in the lumbar region. The lumbar and median sacral arteries frequently become obliterated by atheromatous lesions during adult life, and this can contribute to development of disc degeneration and low back pain [3]. Lumbar artery aneurysms are uncommon lesions that present secondary to vessel injury, and they can rupture due to blunt trauma on the flanks [4]. Despite their small size and retroperitoneal location, these lesions are potentially lethal once they rupture because they can cause shock due to blood loss [5]. Lumbar arteries can provide a collateral source of blood supply to the spinal cord [6]. Interruption of blood flow in the lumbar arteries may be responsible for at least some cases of postoperative paraplegia in surgery for thoracoabdominal aneurysms [7]. 

In endovascular grafting of abdominal aortic aneurysms, lumbar arteries in the aneurysm sac are not ligated, and therefore these can potentially transmit pressure and cause a rupture of the sac [8]. Endoleaks are a major complication of endovascular abdominal aortic aneurysm repair [9]. There are variations in the lumbar vasculature in the retroperitoneum [10]. According to these researchers, lumbar arteries follow a fairly regular pattern. Individual variations occur mainly in the number of arteries arising from the abdominal aorta (range 2–4). Three paired vessels arising posteriorly below the infrarenal aorta were the commonest [10]. The previous studies, which were done on variations in the lumbar vasculature, do not include the South East Asian population. In South East Asia, there are no comprehensive studies carried out and published with regard to variations in the anatomy of the lumbar vasculature. Therefore there is a necessity to carry out such a study. This study was carried out with a view to describe the anatomy of lumbar arteries of adults with regard to their numbers, origin, and the pathway.

## 2. Materials and Methods

A descriptive study was done in the Departments of Anatomy, Faculty of Medicine, University of Kelaniya, Sri Lanka. Hundred and nine Sri Lankan adult human cadavers were dissected to study the variations of lumbar arteries. All the cadavers used for the study were people from Sri Lanka who have been living in Sri Lanka for generations. All the cadavers belong to the Sinhalese ethnicity.

**Figure 1:**
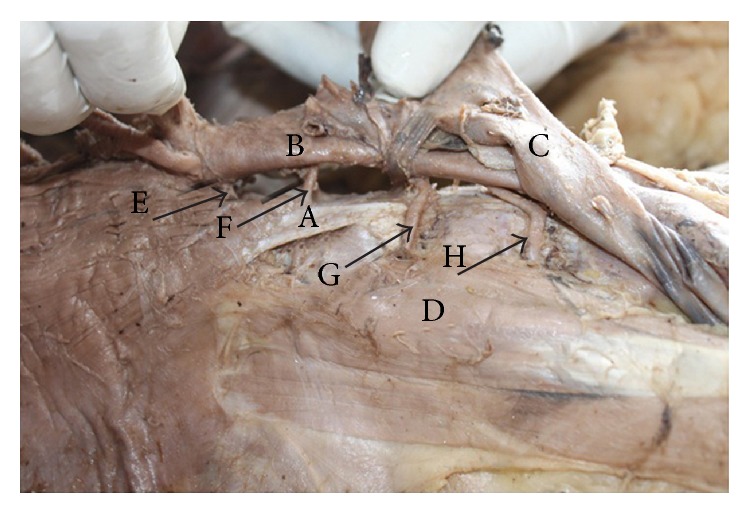
A cadaver demonstrating the posterior abdominal wall structures of the right side of the body. In this specimen, the inferior vena cava has been dissected and folded to the left side of the body to demonstrate the structures passing posterior to it. The first and second lumbar arteries pass posterior to the right crus of the diaphragm, and the third lumbar artery passes anterior to the right crus of the diaphragm. A: right crus of the diaphragm. B: abdominal aorta. C: inferior vena cava folded onto the left side. D: psoas major muscle. E: first lumbar artery. F: second lumbar artery. G: third lumbar artery. H: fourth lumbar artery.

The lumbar arteries were cleared from their origin at the abdominal aorta up to the point of their passing deep to the tendinous arches of the psoas major muscle. Since the lumbar arteries arise from the posterior aspect of the abdominal aorta and then pass laterally on the vertebral bodies to visualize the lumbar arteries, their pathway on the right side had to dissect and partially fold the inferior vena cava to the left side (Figure 1). Thereafter clearing the fascia was able to visualize the lumbar arteries, vertebral bodies, intervertebral discs, right crus of the diaphragm, and the tendinous arches of the psoas. On the left side by clearing the fascia managed to expose the lumbar arteries, vertebral bodies, intervertebral discs, left crus of the diaphragm, and the tendinous arches of the psoas. To identify the lumbo sacral junction passed the index finger posterior to the abdominal aorta and then moved the finger along the vertebral bodies towards the pelvis. Once the lumbosacral junction was identified, counting the vertebral bodies upwards from that junction was able to identify and number the vertebral bodies. The lumbar arteries that arose opposite the vertebral bodies 1st, 2nd, 3rd, and 4th vertebral bodies were named first, second, third, and fourth lumbar arteries, respectively. When changes were detected in the origin, number, and course from what is described by [1], they were noted and photographed. Studying and photographing the origin of lumbar arteries from the posterior aspect of abdominal aorta, the abdominal aorta was dissected out of the cadaver and then the posterior aspect was photographed. Since the lumbar arteries are narrow arteries, to make them more marked from the background they were painted in red colour. 

## 3. Results

**Figure 2:**
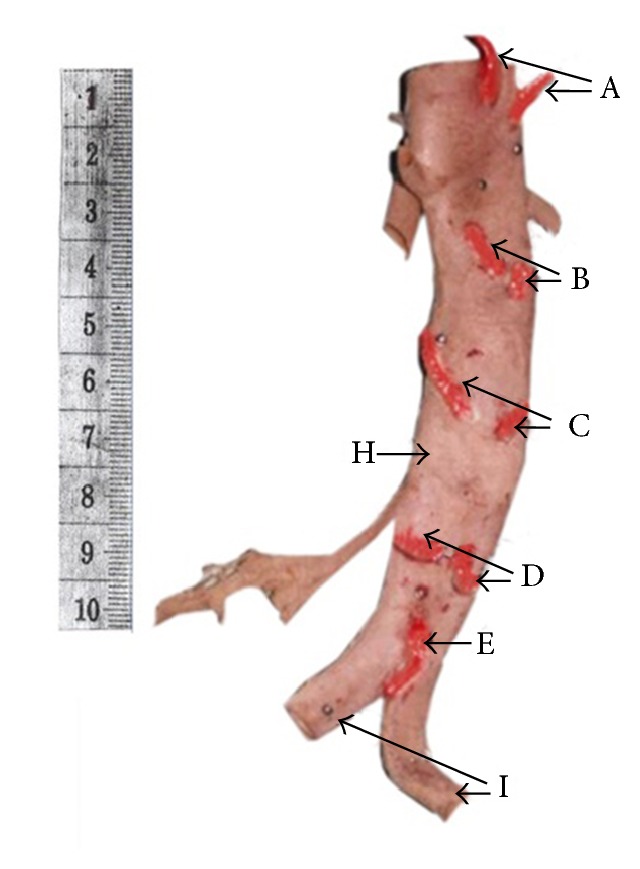
The posterior aspect of a fixed abdominal aorta demonstrating four pairs of lumbar arteries and median sacral artery arising from individual stems. The lumbar arteries and median sacral artery are painted in red for clear identification. A ten-centimeter long scale is kept on the side of the specimen to indicate the size. A: first pair of lumbar arteries. B: second pair of lumbar arteries. C: third pair of lumbar arteries. D: fourth pair of lumbar arteries. E: median sacral artery. H: abdominal aorta. I: common iliac arteries.

Hundred and nine cadavers were dissected of which sixty-two were males (56.9%) and forty-seven were females (43.1%). Their ages ranged from 43 to 90 years. The mean age was 70.1 years, and the standard deviation was 13.6 years. In majority of cadavers (84%), there were four pairs of lumbar arteries and a median sacral artery, and all of them arose from the abdominal aorta from individual stems (Figure 2). However there were many variations with regard to the numbers, origin, and the pathway.

The number of arteries on the left side varied from 3 to 5. Mode 4 and the number of arteries on the right side varied from 3 to 4. 

**Figure 3:**
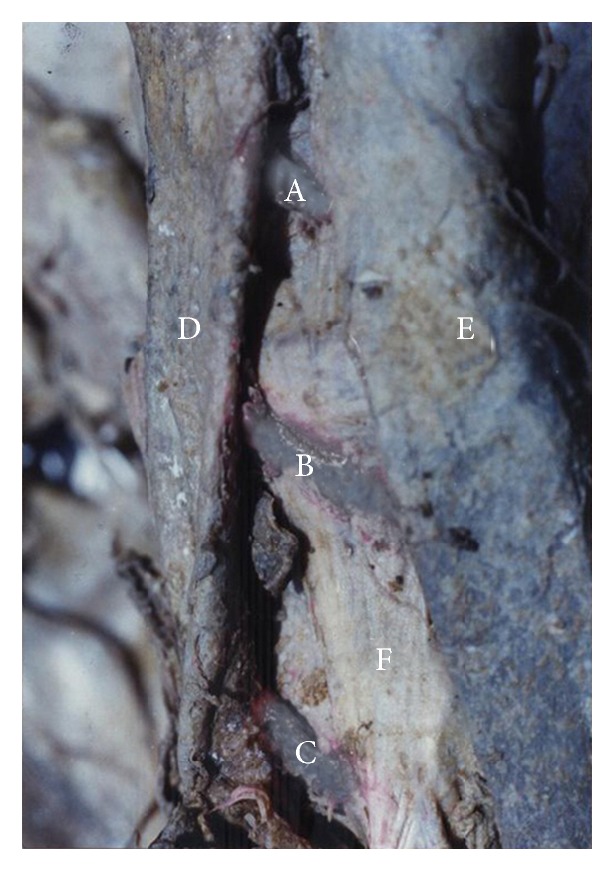
Posterior abdominal wall of a cadaver demonstrating first and second lumbar arteries passing anterior to the right crus of the diaphragm and third lumbar artery passing posterior to the right crus of the diaphragm. A: right first lumbar artery. B: right second lumbar artery. C: right third lumbar artery. D: inferior vena cava. E: abdominal aorta. F: right crus of the diaphragm.

The first pair of lumbar arteries was present in 99% of cadavers. Of these, in one cadaver, the first pair of lumbar arteries originated from a common stem. The first lumbar artery passed anterior to the right crus of the diaphragm in 7% of the cadavers.  Figure 3 demonstrates the first and second lumbar arteries on the right side of the abdominal aorta passing anterior to the right crus of the diaphragm and the third lumbar artery passing posterior to the right crus of the diaphragm.

The second pair of lumbar arteries was present in 99% of cadavers. Of these, in 2% of cadavers, the second pair of lumbar arteries arose from a common stem. In 8% of the cadavers, the second lumbar artery passed anterior to the right crus of the diaphragm (Figure 3). 

The third pair of lumbar arteries was present in 98% of cadavers. Of these, in 3% of cadavers, the third pair of lumbar arteries arose from a common stem. In 13% of cadavers, the third lumbar artery passed posterior to the right crus of the diaphragm (Figure 3). 

**Figure 4:**
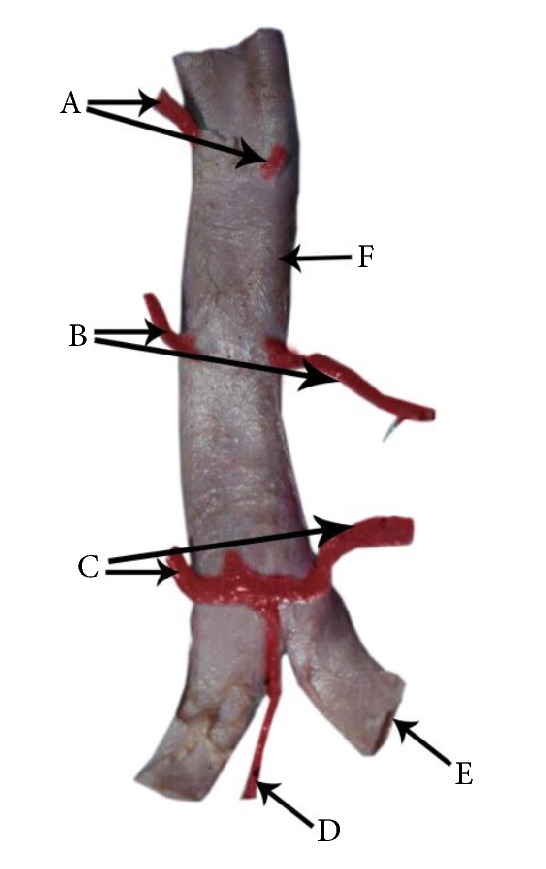
A posterior aspect of a fixed abdominal aorta demonstrating the fourth pair of lumbar arteries and the median sacral artery arising from a common stem. The first pair of lumbar arteries was absent. The lumbar arteries and the median sacral artery are painted in red. A: second pair of lumbar arteries. B: third pair of lumbar arteries. C: fourth pair of lumbar arteries. D: median sacral artery. E: right common iliac artery. F: abdominal aorta.

The fourth pair of lumbar arteries was present in 83% of cadavers. Of these, in 11% of cadavers, the fourth pair of lumbar arteries arose from a common stem.  Figure 4 demonstrates the fourth pair of lumbar arteries and the median sacral artery arising from a common stem from the abdominal aorta. 

**Figure 5:**
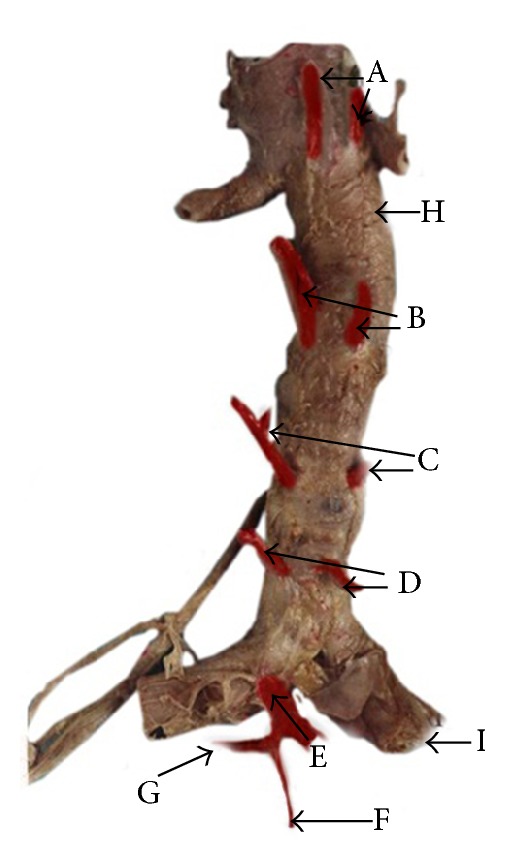
The posterior aspect of a fixed abdominal aorta demonstrating the fifth pair of lumbar arteries and the median sacral artery arising from a common stem from the left common iliac artery. The lumbar arteries and the median sacral artery are painted in red. A: first pair of lumbar arteries. B: second pair of lumbar arteries. C: third pair of lumbar arteries. D: fourth pair of lumbar arteries. E: right fifth lumbar artery. F: median sacral artery. G: left fifth lumbar artery. H: abdominal aorta. I: right common iliac artery.

The fifth pair of lumbar arteries was present only in one cadaver. In that cadaver, the fifth pair of lumbar arteries and the median sacral artery arose from a common stem from the left iliac artery (Figure 5). 

The first pair of lumbar arteries was present in 99% of cadavers, and of them only in 1% of cadavers it did arise from a common stem. The fourth pair of lumbar arteries was present only in 83% of cadavers, and of them in 11% of cadavers it did arise from a common stem. The fifth pair of lumbar arteries was present only in one cadaver, and in that cadaver it arose from a common stem. The lumbar arteries at the lower end of the aorta showed more variations compared to the lumbar arteries at the upper end of the aorta (Table 1). When both lumbar arteries arise from a common stem, the chance of both arteries getting obstructed together by atheromatous lesions is higher and that could give rise to problems such as low-back pain. 

## 4. Discussion

In the present study, there were variations with regard to the total number of lumbar arteries, origin of lumbar arteries, and the pathway of lumbar arteries.

In the current study, the total number of lumbar arteries varied from 3 to 5 pairs. The first three lumbar arteries were almost always present (the first two in 99% and the third in 98% of cadavers). The fourth lumbar artery was present in 83% of the cadavers. There was a fifth lumbar artery in one of the cadavers. The number of lumbar arteries arising from the abdominal aorta decreased from above downwards along the length of abdominal aorta in this study. 

According to Baniel et al., the lumbar arteries followed a fairly regular pattern and individual variations were seen mainly in the total number of lumbar arteries arising from the aorta (range 2–4) [10]. 

In the present study, some pairs of lumbar arteries arose from a common stem from the abdominal aorta. The first pair of lumbar arteries and the second pair of lumbar arteries originated from a common stem in 1% and 2% of cadavers, respectively. The third pair of lumbar arteries and the fourth pair of lumbar arteries arose from a common stem in 3% and 11% of cadavers, respectively. The fifth pair of lumbar arteries was present only in one cadaver, and in that the arteries arose from a common stem (Figure 5). From above downward along the length of the abdominal aorta, the number of arteries arising from a common stem increased. No previous Sri Lankan studies have described these changes. According to Young, the opposite lumbar arteries may arise from a common stem due to the fusion of their origins, or they may arise separately from the aorta at the same level [11]. In the present study the majority of arteries arose from individual stems, and they arose at the same level (Figure 2). 

Low backache can occur due to poor vascular supply to back muscles [12]. Kauppila stated that disc degeneration can occur due to atheromatous lesions of lumbar arteries obstructing the blood flow in the lumbar arteries [3]. According to Ratcliffe, there are connections between the branches of lumbar vessels of the right and left sides [13]. Therefore if there is an obstruction of one artery, the other artery can assist, but when both arteries arise from a common stem both can get obstructed and cause clinical problems such as low backache. In addition to the main blood supply, there are anastomoses in the lumbar region [13]. If only the anastomosis is functioning while the main blood supply is obstructed, there can be clinical problems such as low backache and disc degeneration developing as a result [3]. Mckenzie reported that lower back problems are more common than upper back problems [14]. In the current study, there were a lesser number of lumbar arteries in the lower part of the abdominal aorta, compared with the upper part of the abdominal aorta and more lumbar arteries arose from common stems in the lower part of the abdominal aorta compared with the upper part of the abdominal aorta. Although some previous studies have been described about lumbar arteries arising from common stems, the clinical importance of such findings has not been described. 

In our study, in one cadaver the fourth lumbar artery arose from a common stem with the median sacral artery, and in another cadaver the fifth lumbar artery arose from a common stem with the median sacral artery. According to Garg, a fifth smaller pair occasionally may arise from the median sacral artery [15]. The site of origin of these arteries and their pathway were similar to lumbar arteries. However they may not be lumbar arteries but may be branches of the median sacral artery. 

**Figure 6:**
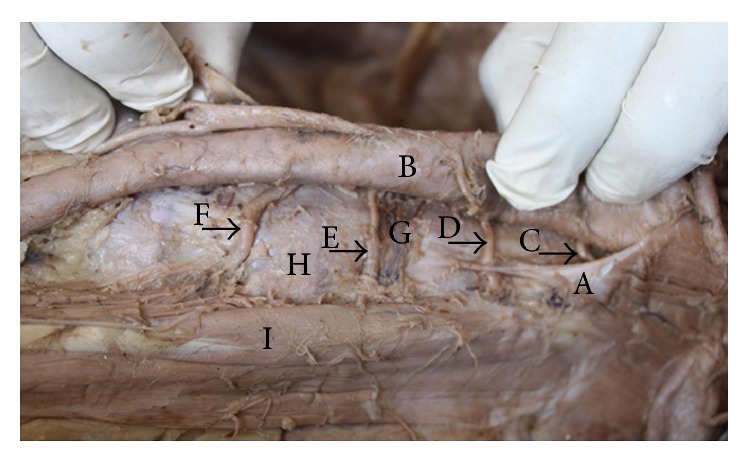
A cadaver demonstrating the posterior abdominal wall structures of the left side of the body. In this specimen, the first and second lumbar arteries on the left side of the body pass posterior to the left crus of the diaphragm. This specimen also demonstrates that the third lumbar artery passes laterally on the vertebral body close to the intervertebral disc. A: left crus of the diaphragm. B: abdominal aorta. C: first lumbar artery. D: second lumbar artery. E: third lumbar artery. F: fourth lumbar artery. G: lumbar intervertebral disc between second and third lumbar vertebral bodies. H: third lumbar vertebral body. I: psoas major muscle.

According to Standing, upper two lumbar arteries on the right side and the first lumbar artery on the left side pass posterior to the crura of the diaphragm [1]. However they do not state the relationship of the right third lumbar artery to the right crura and the left second lumbar artery to the left crura. According to Garg, only the upper lumbar arteries on the both sides pass posterior to the crura [15]. They do not specify the exact number of lumbar arteries that pass posterior to the crura on right and left sides. In our study, in 13% of the cadavers the third lumbar artery on the right side passed posterior to the right crus of the diaphragm (Figure 3), and the left second lumbar artery passed posterior to the left crus of the diaphragm in 100% of cadavers (Figure 6). In this study in few cadavers the first (7%) and second (8%) lumbar arteries on the right side passed anterior to the right crus of the diaphragm (Figure 3) and none of the left first and second lumbar arteries passed anterior to the left crus of the diaphragm.

Surgical repair of paraoesophageal hernias involves closing of defects in the crura of the diaphragm [16]. Therefore lumbar arteries, which pass anterior to the crura of the diaphragm, are at a greater risk of damage than the ones passing posterior to the crura of the diaphragm during surgery involving crura of the diaphragm. 

## 5. Conclusions

In 12% of cadavers lumbar arteries arose from a common stem. The fourth lumbar artery was the commonest (11%) artery that arose from a common stem, and the first lumbar artery was the least common (1%) artery that arose from a common stem. The first and second lumbar arteries passed anterior to the right side crura of the diaphragm in 7% and 8% of the cadavers, respectively. Some variations described in this study have not been described in many previous literature. Having knowledge of such anatomical variations in individuals will help surgeons who perform surgery in the retroperitoneal region and the diaphragmatic region. 

## Figures and Tables

**Table 1:** Anatomical features of all five lumbar arteries (*n* = 109).

Artery	Arteries Present		Arose from a Common Stem		Passed Anterior to Right crus		Passed Anterior to Left crus	
	No	%	No	%	No	%	No	%
1st	108	99	1	1	8	7	0	0
2nd	108	99	2	2	9	8	0	0
3rd	107	98	3	3	93	87	—	—
4th	91	83	10	11	—	—	—	—
5th	1	1	1	100	—	—	—	—

No—indicates the number of cadavers exhibiting the particular feature.
